# Increased Prevalence of Chronic Lymphocytic Thyroiditis in Korean Patients with Papillary Thyroid Cancer

**DOI:** 10.1371/journal.pone.0099054

**Published:** 2014-06-13

**Authors:** Chang-Mo Oh, Sohee Park, Joo Young Lee, Young-Joo Won, Aesun Shin, Hyun-Joo Kong, Kui-Sun Choi, You Jin Lee, Ki- Wook Chung, Kyu-Won Jung

**Affiliations:** 1 National Cancer Control Institute, National Cancer Center, Goyang, Korea; 2 Department of Epidemiology and Health Promotion, Yonsei University, Graduate School of Public Health, Seoul, Korea; 3 National Emergency Medical Center, National Medical Center, Seoul, Korea; 4 Department of Preventive Medicine, Seoul National University, College of Medicine, Seoul, Korea; 5 Center for Thyroid Cancer, National Cancer Center, Goyang, Korea; 6 Division of endocrine surgery, Department of surgery, Asan Medical Center, Seoul, Korea; National Health Research Institutes, Taiwan

## Abstract

**Background:**

In recent years, some reports have suggested that papillary thyroid cancers are more frequently associated with lymphocytic thyroiditis or Hashimoto's thyroiditis. This study investigated a potential increase in the prevalence of chronic lymphocytic thyroiditis among papillary thyroid cancer patients.

**Materials and Methods:**

We used national epidemiological survey data on thyroid cancer patients diagnosed in 1999, 2005, and 2008. A retrospective medical record survey was conducted by representative sampling of a national cancer incidence database. The analysis included 5,378 papillary thyroid cancer patients aged 20–79 years. We calculated the age-standardized prevalence and age-adjusted prevalence ratios using a binomial regression model with a log link for the prevalence of chronic lymphocytic thyroiditis among papillary thyroid cancer patients by sex for each year.

**Results:**

The prevalence of chronic lymphocytic thyroiditis among papillary thyroid cancer patients was 4.0% and 12.8% for men and women in 1999, 6.5% and 24.6% in 2005, and 10.7% and 27.6% in 2008, respectively. Between 1999 and 2008, the age-standardized prevalence of chronic lymphocytic thyroiditis increased 4.1-fold in male patients and 2.0-fold in female patients with papillary thyroid cancer. The prevalence of other thyroid diseases, however, did not increase in either gender.

**Conclusions:**

Among Korean papillary thyroid cancer patients, the prevalence of chronic lymphocytic thyroiditis increased between 1999 and 2008, whereas the prevalence of other thyroid disorders did not change.

## Introduction

The incidence of thyroid cancer has increased rapidly in most developed countries [1]. In Korea, which has the highest incidence of thyroid cancer in the world [2], the incidence also increased rapidly between 1999 and 2010 from 2.1 to 18.3 per 100,000 in men and from 10.4 to 87.4 per 100,000 in women [3]. Papillary thyroid cancer (PTC) is the most common subtype of thyroid cancer, accounting for 90% of all thyroid cancer occurrences [4]. The increasing incidence of PTC cannot be explained simply by an increase in its detection because the incidence of thyroid cancer among children and adolescents, who are not expected to be exposed to screening, has also increased [5]. Additionally, increases were observed not only in thyroid microcarcinomas (248%) but also in carcinomas >5 cm (222%) from 1988–1991 to 2003–2005 among white women in the United States, suggesting that changes in risk factors for thyroid cancer may be responsible for the increase in incidence [6].

There have been several recent reports of increasing trends in thyroid disorders. In Scotland, the overall prevalence of thyroid dysfunction increased from 2.3% in 1994 to 3.8% in 2001 [7]. In Sicily, the number of new patients with Hashimoto's thyroiditis increased from 35 to 484 (138%) between 1975 and 2005 [8]. In Korea, the prevalence of thyroid disorders increased from 1.0% in 1998 to 3.9% in 2010 among people ≥30 years of age [9]. The parallel increases in the incidences of PTC and thyroid disorders suggest a close association between PTC and thyroid disorders [10], [11]. Indeed, concordant increasing trends in frequency distribution of thyroid cancer and Hashimoto's thyroiditis were observed from 1972 to 1991 in the northeastern regions of Kazakhstan adjacent to the nuclear bomb test site [12].

Some epidemiologic studies reported that the proportion of lymphoid thyroiditis or Hashimoto's thyroiditis with PTC has increased in Argentina [13], Italy [14], [15], China [16] and the United States [17]. These pathologic changes in PTC were consistent in various regions and countries. However, no population-based study has addressed this issue. Therefore, we used representative population-based data to investigate whether there is an increase in the prevalence of chronic lymphocytic thyroiditis in Korean PTC patients.

## Materials and Methods

### Data sources

The Korea Central Cancer Registry (KCCR), established by the Korean Ministry of Health and Welfare, was founded in 1980 as a nationwide, hospital-based cancer registry program, and it has been expanding the national population-based cancer registry since 1999. The KCCR generated the Korea National Cancer Incidence Database (KNCI DB), which includes nationwide cancer incidences between 1999 and 2010. Detailed information about the KCCR and KNCI DB has been described previously [3], [18].

The KCCR conducted the National Epidemiologic Survey of Thyroid cancer (NEST) in 2011 to investigate secular trends in the clinicopathologic features of Korean thyroid cancer patients. The NEST selected a sample of thyroid cancer patients diagnosed in 1999, 2005, and 2008 from KNCI DB. The 2005 data were sampled as a midway point between 1999 and 2008 because thyroid cancer among women became the most frequent cancer site since 2005.

The NEST used a 2-stage random sampling procedure to secure the nationwide sample of thyroid cancer patients in Korea. Stage 1 involved the selection of particular hospitals in geographic areas using the KNCI DB, and stage 2 involved the selection of patients within each hospital with probability proportionate to size. Considering a 10% dropout rate, the sampling proportions used were 33% for 1999, 22% for 2005, and 11% for 2008.

A total of 6,846 patients were sampled: 1,103 in 1999, 2,785 in 2005, and 2,958 in 2008. Of 24 sampled hospitals, 2 declined to participate (a total 1,045 cases for 3 years).

**Figure 1:**
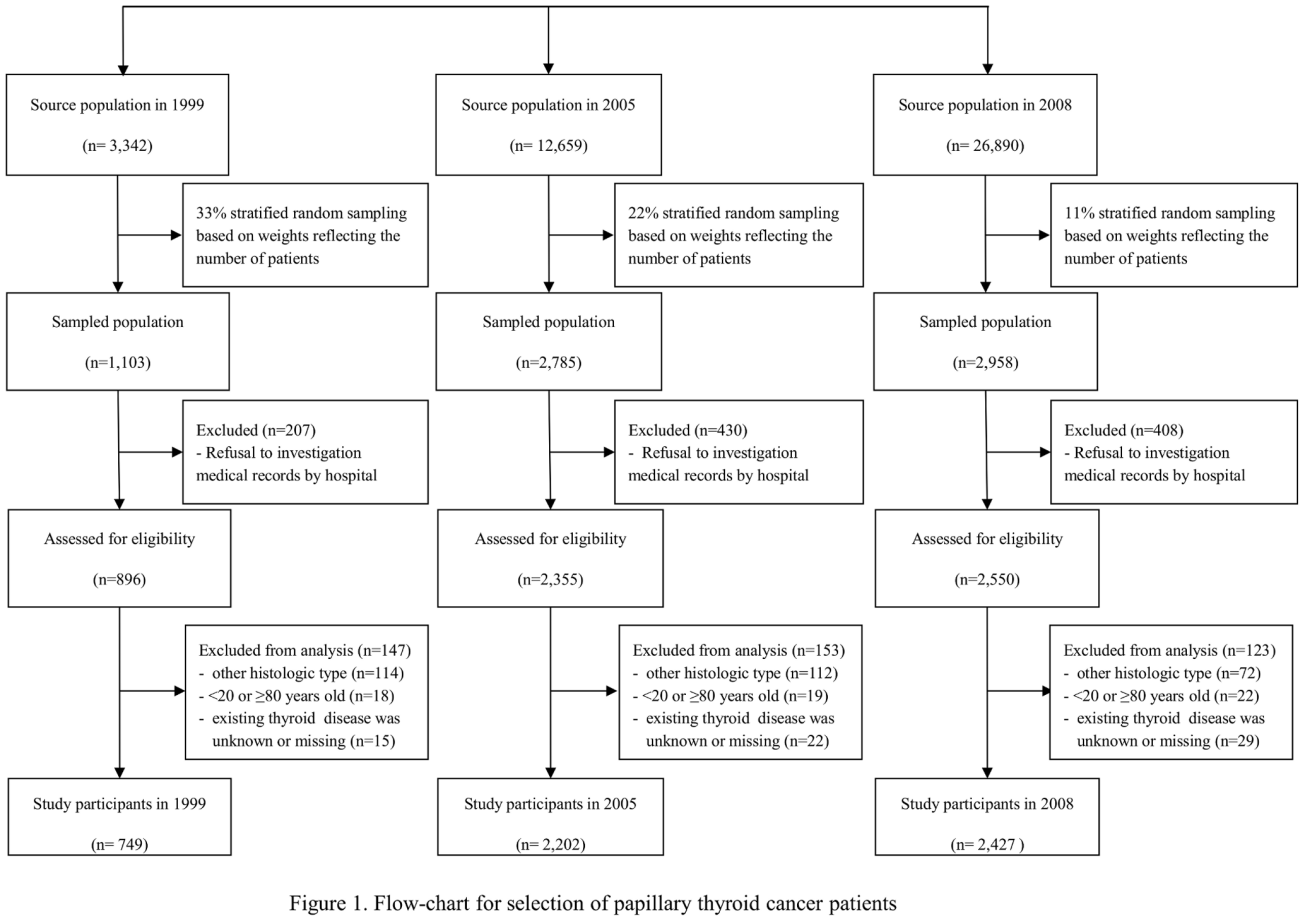
Flow chart for the selection of papillary thyroid cancer patients.

In this study, we restricted the PTC patients to those between the ages of 20 and 79 years. We excluded patients aged <20 or ≥80 years because of small numbers. PTC was classified by International Classification of Diseases O-3 codes 8050, 8260, 8340–8344, 8350, and 8450–8460 [19]. Of the remaining 5,801 thyroid patients, 423 (7.3%) were excluded at least one of following reasons: 298 were not classified as having PTC, 43 were <20 years of age and 16 were ≥80 years of age, and 66 did not have information about coexisting thyroid diseases. Therefore, 5,378 patients were included in the final analysis (Figure 1).

We collected information on age at diagnosis, gender, region, date of diagnosis, histologic type, initial treatment method, tumor size, extrathyroidal invasion, multifocality, tumor-node-metastasis (TNM) stage of thyroid cancer, and coexisting thyroid diseases as confirmed by surgical pathology analysis. The TNM stage was classified according to guidelines of the 2002 American Joint Committee on Cancer [20]. Coexisting thyroid diseases were diagnosed based on the histologic report. Reactive thyroiditis, lymphocytic thyroiditis, and Hashimoto's thyroiditis were all categorized as chronic lymphocytic thyroiditis according to the Bethesda System for reporting thyroid cytopathology [21]. Other benign disorders, including adenomatous goiter, hyperplasia, or granulomatous thyroiditis, were categorized as other thyroid disorders. Extrathyroid invasion was defined as a tumor extension beyond the thyroid capsule [22]. Multifocality was defined as a PTC case which had ≥2 foci in thyroidectomy specimen [23]. All data were collected by retrospective chart review and patient records were anonymized and de-identified prior to analysis.

### Statistical analysis

The baseline study characteristics are presented as mean (SE) for continuous variables and as the percentage (SE) for categorical variables. A linear regression model using “PROC SURVEYREG” in SAS 9.2 (SAS Institute Inc., Cary, NC) was used to test for linear trends over time and the Rao-Scott chi-square test using “PROC SURVEYFREQ” in SAS 9.2 (SAS Institute Inc., Cary, NC) were used. We calculated the age-standardized prevalence and the 95% confidence intervals (CIs) for chronic lymphocytic thyroiditis by gender and year using direct standardization with the world standard population (using “PROC SURVEYREG” in SAS 9.2). We also used binomial regression with a log link to calculate the age-adjusted prevalence ratios and 95% CIs for chronic lymphocytic thyroiditis by gender [24]. Additionally, we considered years as a continuous variable when testing for linear trends in the age-adjusted prevalence ratio of chronic lymphocytic thyroiditis by sex. A *p*<0.05 was considered statistically significant.

## Results

Table 1 shows the baseline characteristics of the study sample according to year of diagnosis. The mean age of patients was 47.2 years, and 84.3% were women. There were no significant differences in the age distribution by year (*p* = 0.57). The tumor size in PTC patients decreased from 19.0 (0.8) mm in 1999 to 10.2 (0.3) mm in 2008 (*p*<0.001). The proportion of extrathyroidal invasion of PTC patients has significantly increased from 44.9% in 1999 to 51.2% in 2008 (*p*<0.001). Although it was not significant statistically, the proportion of multifocality of PTC showed a decreasing trend. The proportion of patients with lymph node involvement (*p* = 0.001) and distribution of TNM stage differed over time (*p*<0.001), respectively. Especially, proportion of stage III papillary thyroid cancer has increased from 13.4% in 1999 to 25.5% in 2008. Although it was not significant statistically, the proportion of distant metastasis also decreased from 1.0% in 1999 to 0.0% in 2008.

**Table 1:** Baseline characteristics of study participants of papillary thyroid cancer across the year.

Variables	Total		Year			*p* for trend
			1999		2005		2008		
N	5378		749		2202		2427		
Age (year)	47.2 (0.3)		46.4 (0.8)		47.4 (0.4)		47.2 (0.4)		0.57*
Sex									0.38†
Men	15.7	(0.8)	16.5	(2.4)	14.1	(1.4)	16.3	(1.1)	
	(n = 808)		(n = 109)		(n = 296)		(n = 403)		
Women	84.3	(0.8)	83.5	(2.4)	85.9	(1.4)	83.7	(1.1)	
	(n = 4570)		(n = 640)		(n = 1906)		(n = 2024)		
Tumor size (mm)	11.5 (0.2)		19.0 (0.8)		12.6 (0.4)		10.2 (0.3)		<0.001*
	(n = 5187)		(n = 675)		(n = 2123)		(n = 2389)		
Extrathyroidal invasion	48.2 (1.2)		44.9 (2.7)		42.4 (1.6)		51.2 (1.6)		<0.001†
missing	(n = 272)		(n = 86)		(n = 116)		(n = 70)		
Multifocal disease	28.8 (0.9)		30.6 (2.7)		30.0 (1.4)		28.1 (1.2)		0.42†
missing	(n = 154)		(n = 57)		(n = 63)		(n = 34)		
Lymph nodes involvement									0.001†
None	56.0	(1.2)	42.3	(3.1)	58.2	(1.7)	56.2	(1.6)	
	(n = 2343)		(n = 234)		(n = 960)		(n = 1149)		
Regional lymph node metastasis	44.0	(1.2)	57.7	(3.1)	41.8	(1.7)	43.8	(1.6)	
	(n = 1970)		(n = 291)		(n = 776)		(n = 903)		
Unknown	(n = 1065)		(n = 224)		(n = 466)		(n = 375)		
Metastasis									
No	99.8	(0.1)	99.0	(0.5)	99.6	(0.2)	100.0	(0.0)	
	(n = 5108)		(n = 678)		(n = 2099)		(n = 2331)		
Yes	0.2	(0.1)	1.0	(0.5)	0.4	(0.2)	0.0	(0.0)	
	(n = 16)		(n = 7)		(n = 9)		(n = 0)		
missing	(n = 254)		(n = 64)		(n = 96)		(n = 96)		
TNM stage									<0.001†
StageI	68.4	(1.0)	69.4	(2.8)	71.1	(1.5)	67.1	(1.4)	
	(n = 2901)		(n = 385)		(n = 1199)		(n = 1317)		
StageII	0.7	(0.3)	2.1	(0.7)	1.5	(1.0)	0.3	(0.1)	
	(n = 32)		(n = 12)		(n = 13)		(n = 7)		
StageIII	22.8	(0.8)	13.4	(1.5)	18.4	(1.1)	25.5	(1.2)	
	(n = 1014)		(n = 86)		(n = 368)		(n = 560)		
StageIV	8.1	(0.7)	15.1	(2.6)	8.9	(0.8)	7.1	(1.0)	
	(n = 365)		(n = 74)		(n = 160)		(n = 131)		
missing	(n = 1066)		(n = 192)		(n = 462)		(n = 412)		

Data was obtained from national epidemiological survey of thyroid cancer.

Continuous data were expressed as Mean (standard error) using PROC SURVEYMEANS.

Categorical data were expressed as Percentage (standard error) using PROC SURVEYFREQ.

*p-values were calculated excluding missing values by linear regression for continuous variables (“PROC SURVEYREG”).

† *p*-values were calculated excluding missing values by Rao-scott chi-square test (“PROC SURVEYFREQ”).

Table 2 shows the changes in the prevalence of chronic lymphocytic thyroiditis among PTC patients. The age-standardized prevalence of chronic lymphocytic thyroiditis was 3.99% (95% CI: 0.31%–7.66%) in 1999, 6.47% (95% CI: 2.11%–10.83%) in 2005, and 10.74% (95% CI: 6.17%–15.32%) in 2008 for male PTC patients and 12.78% (95% CI: 9.65%–15.91%) in 1999, 24.62% (95% CI: 21.40%–27.84%) in 2005, and 27.61% (95% CI: 24.28%–30.94%) in 2008 for female PTC patients. The age-adjusted prevalence ratio for chronic lymphocytic thyroiditis among male PTC patients increased by 155% between 1999 and 2005 and by 311% between 1999 and 2008 (*p* for trend  = 0.004). In female patients, the prevalence ratios increased by 62% between 1999 and 2005 and by 100% between 1999 and 2008 (*p* for trend <0.001). In addition, lymphocytic thyroiditis is more frequently associated to differentiated thyroid cancer in females respect to male.

**Table 2:** Change in the prevalence of chronic lymphocytic thyroiditis among papillary thyroid cancer patients from 1999 to 2008 in Korea.

Gender	Year	No. of papillary thyroid cancer patients		Prevalence of chronic lymphocytic thyroiditis				*p* for trend*
		Total	lymphocytic thyroiditis	Age-standardized prevalence, %		Age adjusted prevalence ratio		
Men	1999	109	3	3.99	(0.31, 7.66)	1	(Reference)	0.004†
	2005	296	20	6.47	(2.11, 10.83)	2.55	(0.77, 8.41)	
	2008	403	45	10.74	(6.17, 15.32)	4.11	(1.30, 12.97)	
Women	1999	640	88	12.78	(9.65, 15.91)	1	(Reference)	<0.001†
	2005	1906	417	24.62	(21.40, 27.84)	1.62	(1.31, 2.00)	
	2008	2024	549	27.61	(24.28, 30.94)	2.00	(1.63, 2.46)	

Data was obtained from national epidemiological survey of thyroid cancer.

The standard population was defined as the world standard population.

*p for trends were calculated by binomial regression using a log link after adjusting for age.

† *p*<0.05.

Table 3 shows the changes in the prevalence of thyroid diseases other than chronic lymphocytic thyroiditis among PTC patients. Unlike chronic lymphocytic thyroiditis, the age-adjusted prevalence ratio for other thyroid diseases among PTC patients did not increase significantly from 1999 to 2008 in either men or women (*p* for trend  = 0.93 and 0.91, respectively).

**Table 3:** Change in the prevalence of other thyroid disease among papillary thyroid cancer patients from 1999 to 2008 in Korea.

Gender	Year	No. of papillary thyroid cancer patients		Prevalence of other thyroid disease				*p* for trend*
		Total	Other thyroid disease	Age-standardized prevalence, %		Age adjusted prevalence ratio		
Men	1999	109	21	15.65	(7.74, 23.56)	1	(Reference)	0.93
	2005	296	61	14.39	(10.10, 18.68)	1.10	(0.71, 1.71)	
	2008	403	72	15.13	(11.14, 19.13)	0.99	(0.64, 1.55)	
Women	1999	640	139	16.54	(13.23, 19.84)	1	(Reference)	0.91
	2005	1906	409	17.87	(15.49, 20.25)	0.96	(0.81, 1.13)	
	2008	2024	448	20.11	(17.09, 23.15)	1.00	(0.85, 1.18)	

Data was obtained from national epidemiological survey of thyroid cancer.

The standard population was defined as the world standard population.

*p for trends were calculated by binomial regression using a log link after adjusting for age.

† *p*<0.05.

## Discussion

To our best knowledge, this study is the first to investigate temporal changes in the prevalence of chronic lymphocytic thyroiditis among PTC patients through population-based registry data. The study showed that the proportion of PTC patients with chronic lymphocytic thyroiditis increased between 1999 and 2008, whereas the proportion of patients with other thyroid diseases did not change.

Our study also showed consistently that lymphocytic thyroiditis was predominant in female PTC patients. In the hospital based retrospective study, lymphocytic thyroiditis is more frequently associated to differentiated thyroid cancer in females respect to males [25], [26]. In addition, interestingly, proportion of stage III papillary thyroid cancer has increased in our study over time. Increased proportion of stage III papillary thyroid cancer may be attributed to increase in lymph node involvement of PTC. Some studies reported that lymph node invasion was more frequent in PTC patients with thyroiditis than those without thyroiditis [27], [28]. These findings were consistent with our results. Although it was not significant, lymph node invasion was more frequent in PTC patients with lymphocytic thyroiditis (41.3%) than those without lymphocytic thyroiditis (35.7%) in Korea. It may explain that lymph node invasion in stage III PTC patients has increased along with increase in proportion of lymphocytic thyroiditis among PTC patients across the year.

Increasing prevalence of chronic lymphocytic thyroiditis among papillary thyroid cancer patients is consistent with findings in studies of Argentina [13], Italy [14], [15], China [16], and the United States [17]. This consistent epidemiologic evidence suggests that such an increase in PTC with chronic lymphocytic thyroiditis or Hashimoto's thyroiditis seems to be an epidemic phenomenon across multiple countries rather than a change confined to a specific country.

The close association between chronic lymphocytic thyroiditis and PTC is well known. A recent meta-analysis showed that the association between Hashimoto's thyroiditis and PTC is significantly 2.8-fold higher than that between benign thyroid diseases and PTC [29]. In addition, simultaneous increasing trends in frequency distribution of thyroid cancer and Hashimoto's thyroiditis were observed from 1972 to 1991 in the northeastern regions of Kazakhstan adjacent to the nuclear bomb test site [12].

Chronic lymphocytic thyroiditis and PTC may share a possible risk factor, excessive intake of iodine [30], [31]. In general, it has been known that the Korean population has a higher intake of iodine than the western population [32], and the dietary intake of iodine-rich foods, including milk and seaweed, increased from 1969 to 2005 (Figure S1) [33]. Even though the association between excessive iodine intake and PTC remains unclear [34], it has been suggested that high iodine intake could increase the risk of PTC [13], [31]. In Korean adults, a case-control study suggested that thyroid cancer patients have a higher urinary iodine level than normal control patients [32]. Furthermore, a cohort study of Japan identified a positive association between seaweed consumption and the risk of thyroid cancer [35].

Additionally, changes in iodine intake are proposed to be responsible for the increases of chronic lymphocytic thyroiditis in PTC. Previous studies conducted in Argentina [13], Italy [15], and China [16] also reported that the prevalence of lymphoid thyroiditis or Hashimoto's thyroiditis with PTC increased after iodine supplementation. A study in Salta, Argentina, found that the frequency of lymphoid thyroiditis with PTC in women increased after iodine supplementation [13]. A study in Novara, Italy, also reported that the prevalence of coexisting autoimmune thyroiditis increased in thyroid cancer patients from 1997–2005 to 2006–2010 after iodine prophylaxis [15]. A study in Shenyang, China, showed that the proportion of lymphoid thyroiditis with PTC cases in women increased after a salt iodization program [16]. There was a corresponding increasing trend in the intake of iodine-rich foods from 1969 to 2005 in Korea (Figure S1) [33].

An increase in detection is the most important factor that affects the increased incidence in thyroid cancer, as shown in many previous studies [36]–[38]. It may also be attributed to the increase in chronic lymphocytic thyroiditis among the general population. The increasing rate of coexistence of PTC and chronic lymphocytic thyroiditis could also be attributed to change of the level of histological examination and indications for thyroidectomy over time. However, improved detection or change to level of histological examination is not possible to explain fully the increase in thyroid cancer [39], [40]. Indeed, prevalence of other coexisting thyroid diseases in PTC did not increase significantly in the current study. Therefore, it is reasonable to infer that prevalence of other coexisting thyroid diseases in PTC also has increased if the increase in the prevalence of chronic lymphocytic thyroiditis in PTC is due to an increase in detection or change to level of histological examination. Furthermore, previous studies indicated consistently that the proportion of adenoma in thyroid cancer patients did not increase significantly [14]–[16].

The current findings should be interpreted with caution. First, we excluded hospitals that refused to participate and patients who lacked information regarding comorbidities and who were <20 years or ≥80 years of age. The results may show selection bias because of the exclusion criteria, but refusals were determined by hospitals not by patients. Therefore, selection bias due to refusal may be minimal unless characteristics of patients associated with the refusing hospitals differed significantly from among those associated with participating hospitals. Additionally, the patients who had lacked information regarding comorbidities and were <20 or ≥80 years of age were relatively small in number. Second, because of a lack of information on lifestyle, including dietary iodine intake, the potential confounding factors for the association between chronic lymphocytic thyroiditis and PTC were not investigated fully. Third, we could not assess additional clinical and biochemical characteristics of the thyroid disorders, including urinary iodine level, antithyroid autoantibodies, and preoperative levothyroxine levels.

In summary, we show that the prevalence of chronic lymphocytic thyroiditis among PTC patients increased between 1999 and 2008, whereas the prevalence of other coexisting thyroid diseases in PTC did not. The findings are consistent with those of other countries and suggest that changes in risk factors may have affected the increase in the prevalence of PTC and chronic lymphocytic thyroiditis.

## Supporting Information

Figure S1 Secular trends in intake of foods rich in iodine, 1961–2011.
(TIF)
