# Supplementary figures and images for: Increased Prevalence of Chronic Lymphocytic Thyroiditis in Korean Patients with Papillary Thyroid Cancer
Source: PLoS One. 2014 Jun 13;9(6):e99054. doi: 10.1371/journal.pone.0099054 (PMC4057257; doi:10.1371/journal.pone.0099054)

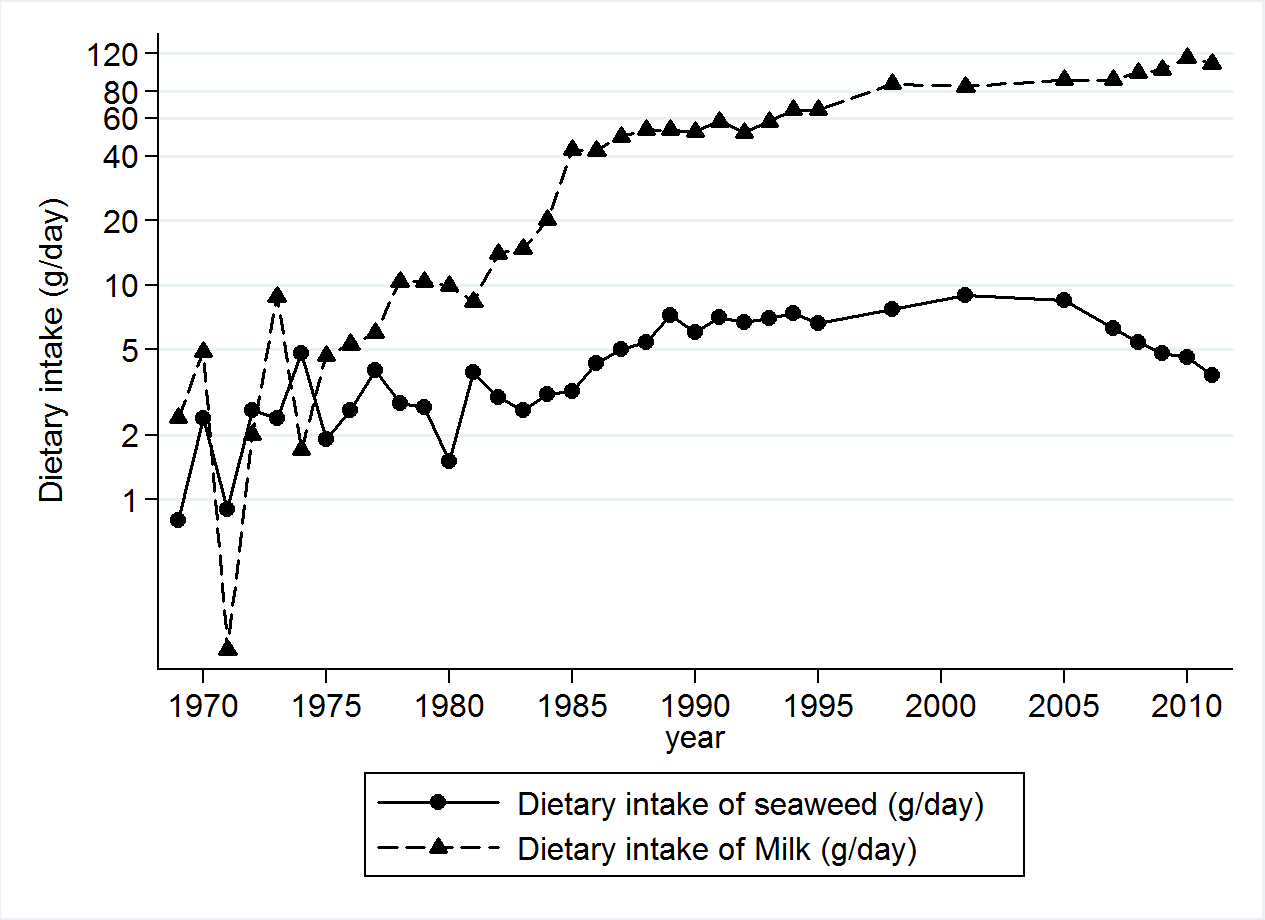

Supplement: Figure S1 — Secular trends in intake of foods rich in iodine, 1961–2011. (TIF) [file pone.0099054.s001.tif]
